# Effect of endometriosis on prognosis of ovarian clear cell carcinoma: a 10-year retrospective study

**DOI:** 10.3389/fonc.2024.1438309

**Published:** 2024-11-15

**Authors:** Yang He, Bo Cao, Yi Huang

**Affiliations:** Department of Gynecological Oncology, Hubei Cancer Hospital, Tongji Medical College, Huazhong University of Science and Technology, Wuhan, China

**Keywords:** ovarian clear cell carcinoma, endometriosis, China, laparoscopic surgery, chemotherapy

## Abstract

**Introduction:**

Endometriosis is considered as a precancerous lesion for OCCC; however its prognostic significance remains controversial. This study aims to evaluate the prognostic significance of endometriosis in patients with ovarian clear cell carcinoma (OCCC) and analyze the impact of other clinical pathological features on prognosis. Additionally, we also assess the role of laparoscopic surgery and chemotherapy in OCCC, hoping to provide evidence for improving the clinical diagnosis and treatment of OCCC.

**Methods:**

A retrospective analysis was conducted on medical records of 105 OCCC patients diagnosed and treated at the Gynecologic Cancer Center of Hubei Cancer Hospital in China from 2013 to 2022. Based on the presence or absence of endometriosis, OCCC patients were divided into two groups: a group with ovarian endometriosis consisting of 44 cases (41.9%) (EC-positive group) and a group without ovarian endometriosis consisting of 61 cases (58.1%)(EC-negative group). Clinical pathological characteristics, progression-free survival (PFS), and overall survival (OS) were compared between the two groups.

**Results:**

There were no statistically significant differences between the two groups in terms of age, CA125, tumor size, FIGO stage, adjuvant chemotherapy regimen, or chemotherapy efficacy (P>0.05). Residual tumor after surgery, staging, site invasion involvement, presence of ascites, positive cytology in ascitic fluid, lymph node metastasis, and chemotherapy efficacy were predictive factors for recurrence among patients with statistical significance (P<0.10); chemotherapy efficacy remained as independent predictors for recurrence (P<0.05); staging and chemotherapy efficacy remained as independent predictors for survival (P<0.05). There was no statistically significant difference observed between both groups regarding OS or PFS.

**Conclusion:**

In this study, co-existing endometriosis was not a prognostic factor for survival in patients with OCCC. The most important predictors of OS and PFS were FIGO stage and chemotherapy sensitivity. The intrinsic link between endometriosis and OCCC requires larger, better-designed prospective studies to draw more definitive conclusions.

## Introduction

As the third most common gynecological malignancy, ovarian cancer is also the leading cause of death among gynecological cancers (1). In 2012, approximately 239,000 new cases of ovarian cancer and 152,000 deaths due to ovarian cancer were reported worldwide (2). OCCC is a unique subtype within epithelial ovarian cancer and accounts for 5% to 10% of all ovarian cancers in North America, and is more common in East Asia (3). Unlike other types of epithelial ovarian cancer, clear cell carcinoma exhibits distinct epidemiological features as well as clinical and pathological characteristics along with unique gene expression profiles and immune microenvironments.

According to epidemiological data, there are significant differences in the incidence rates among different racial populations. According to a report from the United States, the percentage of ovarian clear cell carcinoma (OCCC) in epithelial ovarian cancer is 4.8% among Caucasians, 3.1% among African Americans, and 11.1% among Asians. In Asia, the percentage of OCCC in Japanese epithelial ovarian cancer has significantly increased from 23.4% in 2002 to 29.1% in 2010, and currently remains above 25% (4, 5). Similar incidence rates have also been observed in Taiwan and Singapore. According to data from Beijing Union Medical College Hospital, the proportion of OCCC is 9.7% (6).

According to organizational studies, the occurrence of ovarian clear cell carcinoma (OCCC) may be related to endometriosis, characterized by the presence of abundant glycogen in the cytoplasm (7). Since Sampson first reported the malignant transformation of ovarian endometriotic cysts in 1925, multiple studies have confirmed a significant correlation between OCCC and ovarian endometriosis (EMs) (8). 50% to 74% of OCCC cases are associated with EMs (with ovarian EMs being the most common) (9). Women with endometriosis have three times higher risk of developing OCCC compared to those without endometriosis (10).

In terms of gene expression OCCC is not strongly associated with family history (11). OCCC shares more similarities with Renal Clear Cell Carcinoma (RCCC) rather than other epithelial ovarian cancer subtypes (12). BRCA1/2 germline mutations are rare in OCCC (2.1% to 6%) (13). Its characteristics include frequent ARID1A somatic mutations (approximately 40-62%), overexpression of MDM2, and upregulation of the PI3K-Akt-mTOR-MAPK signaling axis (approximately 33-51%) (14, 23–25). Additionally, deficiency mismatch repair (dMMR) is relatively more common in OCCC (26).

Given the unique clinical behavior of ovarian clear cell carcinoma (OCCC), we need to gain a better understanding of this rare tumor type. Therefore, the purpose of our study was to evaluate the clinical characteristics and outcomes of OCCC patients, assess the prognostic significance of various clinicopathological features, and provide supporting evidence for the clinical diagnosis and treatment of OCCC.

## Methods

### Patient and clinical data

**Figure 1:**
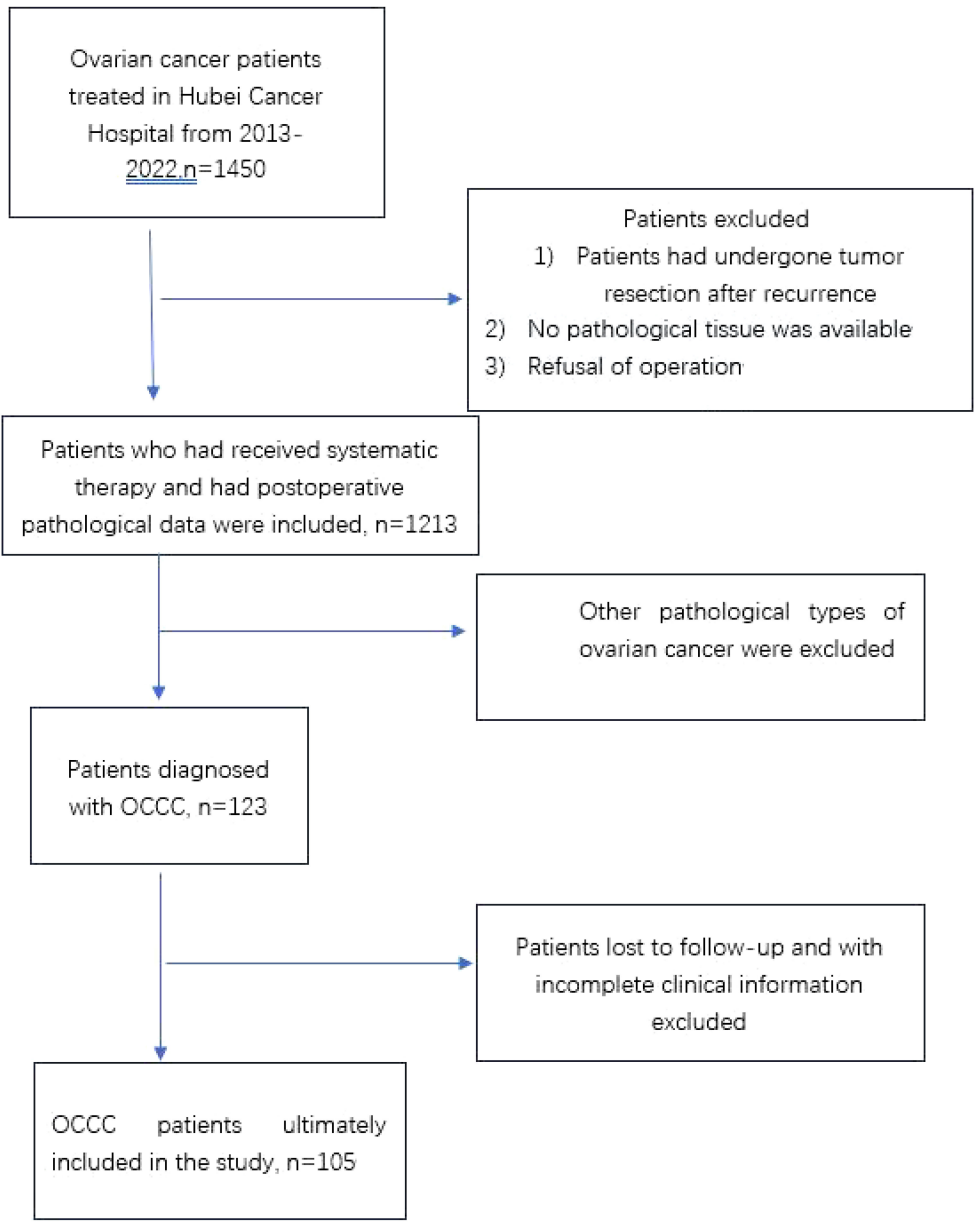
Flowchart of included patients. OCCC, ovarian clear cell carcinoma.

This was a retrospective study conducted on 105 patients diagnosed with primary OCCC at Hubei Cancer Hospital between October 2012 and March 2022. We screened patients according to the prescribed criteria (**Figure 1**), including patients with complete clinical data and follow-up information. The endpoints selected for analysis included PFS and overall survival (OS). All patients underwent surgical treatment, with histopathology confirming pure ovarian clear cell carcinoma. Exclude patients with concomitant malignancies. Patients who were lost to follow-up were excluded from the survival analysis. Patient information was collected from electronic medical records at our center, including age at diagnosis, menopausal status, reproductive history, FIGO stage, CA-125 levels, ovarian tumor size, bilateral involvement of ovaries, ascitic cytology results, surgical approach used, residual tumor status, pathological diagnosis, presence or absence of coexisting endometriosis, lymph node metastasis, chemotherapy regimen, chemotherapy response rate, PFS, total survival, and disease status at last follow-up visit, and evaluated accordingly. The patients were restaged according to FIGO 2014 staging criteria. Presence of endometriosis was checked according to pathologic report from surgery. Pathological diagnosis according to Sampson and Scott’s criteria, (15, 16), (1) co-existence of OCCC and endometriosis in the same ovary; (2) presence of tissue similar to endometrial stroma around the epithelial glands; (3) exclusion of ovarian metastatic disease; (4) presence of benign endometriosis.

### Treatments

All patients underwent either open or laparoscopic surgery. Standard surgical procedures included hysterectomy, bilateral salpingo-oophorectomy, omentectomy, pelvic cytoreductive surgery and/or pelvic and para-aortic lymph node dissection. The majority of women underwent complete surgical staging. Except for two cases that refused postoperative chemotherapy, the rest received platinum-based chemotherapy plus paclitaxel regimen for 3-8 cycles according to NCCN guidelines. Based on the residual tumor after the first cytoreductive surgery, tumor residue was categorized as: no residual tumor (RO), residual tumor measuring 0.1-1 cm (R1), and residual tumor measuring >1 cm. The response to chemotherapy was classified as refractory (progression within one month after chemotherapy), resistant (progression within one to six months after chemotherapy), and sensitive (progression after six months of chemotherapy). Patients followed a similar treatment approach throughout the entire treatment phase and strictly adhered to the current NCCN guidelines for ovarian cancer.

### Statistical analysis

Statistical analysis was performed using SPSS 20.0 for Windows program. The distribution of clinical pathological events was analyzed using chi-square test or Fisher’s exact test. Kaplan-Meier method was used for univariate survival analysis, and Log-rank test was used to compare survival curves. Cox proportional hazards model was used for multivariate analysis to evaluate independent factors affecting survival. A p-value < 0.05 indicated statistical significance.

## Results

We selected 105 cases of OCCC patients admitted to our hospital from January 2012 to March 2022 as research subjects with more than 12 months of follow-up data available. According to the surgical pathological examination results of ovarian cancer, the patients were divided into 44 cases (41.9%) in the EC-positive group and 61 cases (58.1%) in the EC-negative group. Independent sample t-tests were used to analyze age differences between the two groups statistically; however, no significant difference was found in age distribution between them (P>0.05). Chi-square tests were conducted to analyze menopausal status and whether there was residual disease after surgery among other relevant clinical indicators between both groups statistically; it revealed that there were significant differences in menopausal status distribution as well as referral after incomplete surgery in the another hospital between them (P<0.05).The incidence of menopause in the EC-negative group was higher than that in the EC-positive group, while the rate of referral after incomplete surgery in the another hospital was lower in the EC-negative group compared to the EC-positive group (see **Table 1**).

**Table 1:** Presents the analysis of clinical indicators in patients with ovarian clear cell carcinoma in the EC-positive (EC+) group and the EC-negative (EC-) group.

Parameter		EC-	EC+	χ^2^	P
Age		49.05±9.09	45.66±8.43	1.943	0.055
Menopausal	Pre-menopausal	31 (50.8)	33 (75.0)	6.280	0.012
	Post-menopausal	30 (49.2)	11 (25.0)		
Residual disease (RD)	RD: 0	44 (72.1)	37 (84.1)	2.409	0.300
	RD:0~1	13 (21.3)	6 (13.6)		
	RD: >1	4 (6.6)	1 (2.3)		
FIGO stage	Stage I	32 (52.5)	28 (63.6)	1.706	0.636
	Stage II	12 (19.7)	6 (13.6)		
	Stage III	15 (24.6)	8 (18.2)		
	Stage IV	2 (3.3)	2 (4.5)		
Reproduction	No	5 (8.2)	4 (9.1)	0.000	1.000
	Yes	56 (91.8)	40 (90.9)		
CA125 (U/mL)	≤35	13 (21.3)	14 (31.8)	1.477	0.224
	>35	48 (78.7)	30 (68.2)		
Referral to our hospitals after incomplete surgery	No	48 (78.7)	25 (56.8)	5.770	0.016
	Yes	13 (21.3)	19 (43.2)		
Complete staging surgery	No	3 (4.9)	0 (0.0)	0.808	0.369
	Yes	58 (95.1)	44 (100.0)		
Surgical procedure	laparotomy	48 (78.7)	33 (75.0)	0.197	0.657
	Laparoscopic	13 (21.3)	11 (25.0)		
Tumor size	<10cm	10 (16.4)	7 (15.9)	0.004	0.947
	≥10cm	51 (83.6)	37 (84.1)		
Bilaterality	Unilateral	46 (75.4)	39 (88.6)	2.900	0.089
	Bilateral	15 (24.6)	5 (11.4)		
Capsule rapture	No	4 (6.6)	2 (4.5)	0.000	0.990
	Yes	57 (93.4)	42 (95.5)		
Abdominal dropsy	No	35 (57.4)	27 (61.4)	0.168	0.682
	Yes	26 (42.6)	17 (38.6)		
Ascites cytology	Negative	38 (62.3)	33 (75.0)	1.884	0.170
	Positive	23 (37.7)	11 (25.0)		
Lymph node metastasis	No	51 (83.6)	36 (81.8)	0.058	0.810
	Yes	10 (16.4)	8 (18.2)		
Adjuvant chemotherapy	No	2 (3.3)	0 (0.0)	–	0.508
	Yes	59 (96.7)	44 (100.0)		
Chemotherapy regimen	TP	59 (96.7)	43 (97.7)	0.000	1.000
	Other	2 (3.3)	1 (2.3)		
Response to chemotherapy	Sensitive	43 (70.5)	33 (75.0)	0.377	0.828
	Resistant	11 (18.0)	6 (13.6)		
	Refractory	7 (11.5)	5 (11.4)		

χ^2^ Chi-square test, P p-value.

A group with endometriosis (EC-positive group) and a group without endometriosis (EC-negative group).

The asymptomatic survival period, recurrence status, time of death, and survival status of the patients separately, and based on the patients’ recurrence and survival status, the patients were divided into a recurrence group with 59 cases (56.2%) and a non-recurrence group with 46 cases (43.8%), as well as a survival group with 68 cases (64.8%) and a death group with 37 cases (35.2%).

Statistical analysis using univariate Log-Rank test was conducted to examine clinical factors influencing patient recurrence; results indicated that surgical residue, staging, CA125, site of invasion, ascites, cytology of ascitic fluid, lymph node metastasis, chemotherapy effect were associated with patient recurrence outcome and time to recurrence (P<0.10). Cox multivariate survival analysis was performed on statistically significant variables from the univariate analysis. The results indicated that the efficacy of chemotherapy cycles was independent predictors for patient recurrence (P<0.05). Patients who were resistant chemotherapy had a greater risk of recurrence (HR=8.070, 95% C.I.: 4.183-15.569), as shown in **Table 2**.

**Table 2:** Single-factor and multi-factor analysis of factors affecting patient relapse.

	χ^2^	P	HR(95%CI)	χ^2^	P	HR(95%CI)
Age	0.003	0.955	0.999 (0.971-1.028)			
EC	0.339	0.560	0.854 (0.501-1.454)			
Menopausal	0.709	0.400	0.795 (0.466-1.356)			
Residual disease (RD)	38.059	<0.001	3.513 (2.357-5.236)	0.387	0.534	1.227 (0.644-2.336)
FIGO stage	20.627	<0.001	1.851 (1.419-2.415)	2.042	0.153	1.439 (0.873-2.371)
Reproduction	0.072	0.788	1.15 (0.415-3.186)			
CA125U/mL	3.211	0.073	1.825 (0.945-3.526)	1.179	0.278	1.494 (0.724-3.084)
Referral to our hospitals after incomplete surgery	0.502	0.479	0.812 (0.457-1.444)			
Complete staging surgery	2.252	0.133	0.408 (0.127-1.316)			
Surgical procedure	0.000	0.984	1.006 (0.542-1.869)			
Tumor size	0.151	0.698	1.159 (0.549-2.448)			
Bilaterality	8.656	0.003	2.414 (1.342-4.342)	0.248	0.618	0.824 (0.384-1.768)
Capsule rapture	0.160	0.689	1.269 (0.395-4.072)			
Abdominal dropsy	13.885	<0.001	2.715 (1.605-4.59)	1.192	0.275	1.544 (0.708-3.369)
Ascites cytology	17.962	<0.001	3.159 (1.856-5.377)	0.320	0.571	0.763 (0.300-1.944)
Lymph node metastasis	9.573	0.002	2.55 (1.409-4.613)	1.179	0.277	0.610 (0.250-1.489)
Adjuvant chemotherapy	1.563	0.211	0.405 (0.098-1.67)			
Chemotherapy regimen	0.992	0.319	2.056 (0.498-8.489)			
Response to chemotherapy	76.125	<0.001	10.024 (5.973-16.823)	38.788	<0.001	8.070 (4.183-15.569)

HR, hazard ratio; CI, confidence interval; P, p-value; EC, endometriosis; χ^2^, Chi-square test; EC, endometriosis.

**Figure 2:**
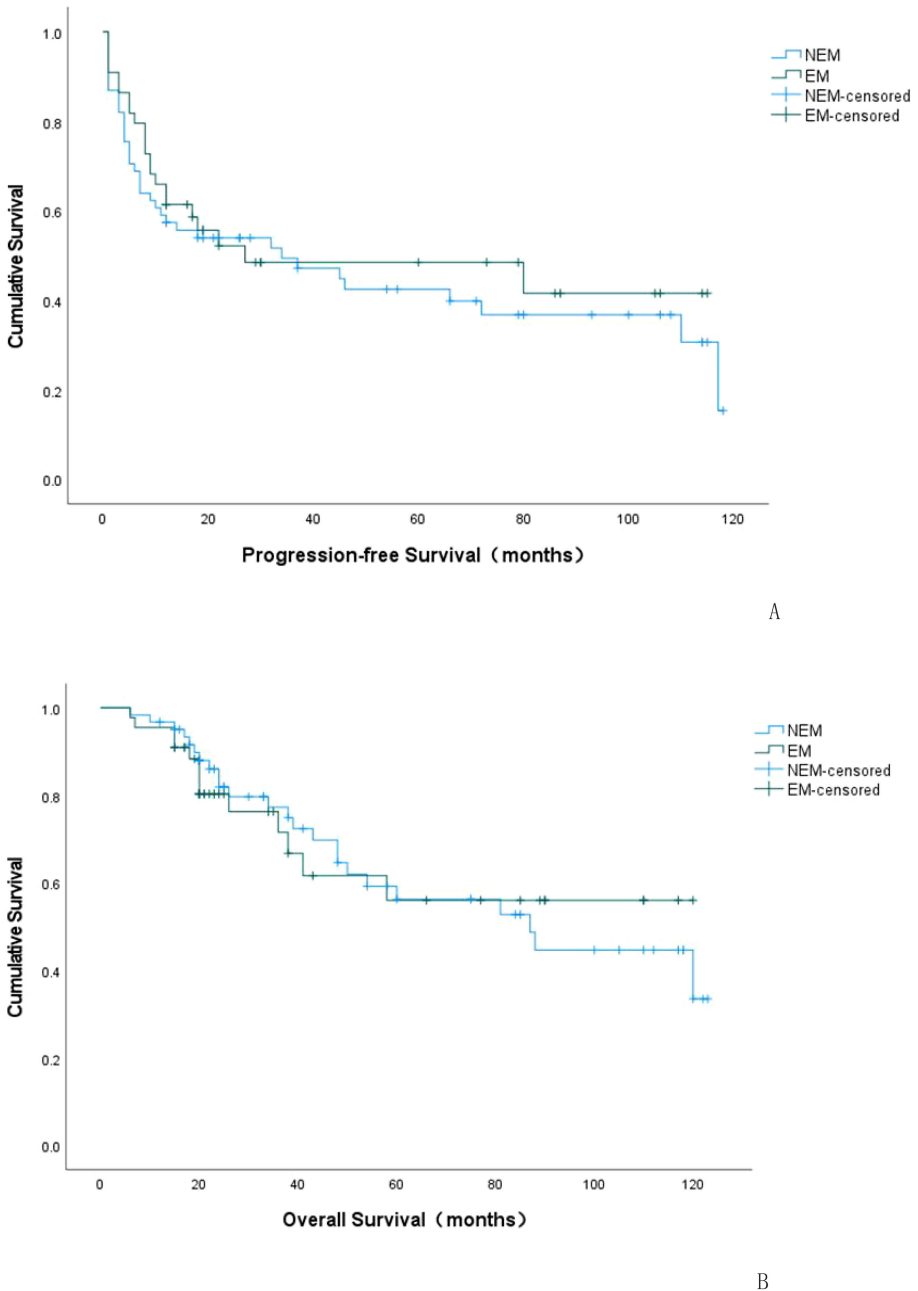
K-M curve of progression-free survival (PFS) in EC-positive group and EC-negative group **(A)**. K-M curve of overall survival (OS) in EC-positive group and EC-negative group **(B)**. NEM: EC-negative group; EM: EC-positive group.

Kaplan-Meier curves were plotted to analyze the survival rates for patients with positive EC status versus negative EC status in terms of their recurrences. According to the survival curve analysis, there was no statistical difference in recurrence status and time between patients with positive EC status and negative EC status (P=0.552). The median time to recurrence for patients with positive EC status was 27 months (95% C.I.: 0-97.67), while it was 34 months for patients with negative EC status (95% C.I.: 0-71.09), as shown in **Table 3** and **Figure 2A**.

**Table 3:** Recurrence status and time analysis in EC-positive group and EC-negative group.

EC	Median recurrence time (m)	S.E. (95% CI)	Log-Rank	P
Negative group	34	18.925 (0-71.093)	0.354	0.552
Positive group	27	36.065 (0-97.688)		
Total	34	11.916 (10.644-57.356)		

S.E, standard error; CI, confidence interval; P, p-value; EC, endometriosis.

Univariate Log-Rank tests were conducted to statistically analyze clinical factors affecting patient survival rates. The results showed that surgical residue, staging, site invasion, ascites, cytology of ascitic fluid, lymph node metastasis, chemotherapy effect were associated with patient survival outcomes and survival time (P<0.10), which are predictive factors affecting patient survival. Cox multivariate survival analysis was conducted to further analyze the statistically significant variables identified in the univariate analysis. The results indicated that staging and chemotherapy efficacy were independent predictive factors for patient recurrence (P<0.05). Patients with stage 3 or 4 had a higher risk of death compared to those with stage 1 or 2 (HR=2.137, 95% C.I.: 1.068-4.276), while patients resistant or not controlled by chemotherapy had a greater risk of death (HR=5.596, 95% C.I.: 2.942-10.643). Please refer to **Table 4** for details.

**Table 4:** Univariate and multivariate analysis of factors influencing patient survival.

	χ^2^	P	HR (95%CI)	χ^2^	P	HR (95%CI)
Age	0.036	0.850	1.004 (0.968-1.041)			
EC	0.008	0.928	0.969 (0.492-1.911)			
Menopausal	0.001	0.982	1.008 (0.525-1.935)			
Residual disease (RD)	29.105	<0.001	3.849 (2.359-6.282)	0.725	0.394	0.685 (0.286-1.637)
FIGO stage	25.359	<0.001	2.343 (1.682-3.264)	4.610	0.032	2.137 (1.068-4.276)
Reproduction	0.005	0.944	0.958 (0.292-3.145)			
CA125U/mL	1.588	0.208	1.699 (0.745-3.874)			
Referral to our hospitals after incomplete surgery	2.492	0.114	0.494 (0.206-1.186)			
Complete staging surgery	0.002	0.960	0.950 (0.129-6.993)			
Surgical procedure	0.096	0.757	0.878 (0.385-2.002)			
Tumor size	0.252	0.616	1.305 (0.461-3.697)			
Bilaterality	9.892	0.002	2.973 (1.508-5.863)	1.577	0.209	1.779 (0.724-4.369)
Capsule rapture	0.499	0.480	2.049 (0.280-15.014)			
Abdominal dropsy	8.029	0.005	2.624 (1.346-5.114)	1.537	0.215	0.445 (0.124-1.600)
Ascites cytology	22.518	<0.001	5.225 (2.639-10.344)	2.672	0.102	2.656 (0.823-8.566)
Lymph node metastasis	9.648	0.002	3.105 (1.519-6.347)	0.141	0.708	0.807 (0.263-2.477)
Adjuvant chemotherapy	1.033	0.309	0.354 (0.048-2.626)			
Chemotherapy regimen	1.240	0.265	3.143 (0.419-23.594)			
Response to chemotherapy	51.186	<0.001	6.932 (4.078-11.781)	27.556	<0.001	5.596 (2.942-10.643)

HR, hazard ratio; CI, confidence interval; P, p-value; EC, endometriosis, χ^2^, Chi-square test; EC, endometriosis.

Kaplan-Meier curves were plotted to depict the survival outcomes between EC-positive group and EC-negative group in terms of overall survival rate among patients with endometriosis-associated OCCC. According to the analysis based on these curves, there was no statistical difference observed in terms of overall survival rate and time between EC-positive group and EC-negative group (P=0.928). The median overall survival period for EC-positive group was estimated at 80.177 months (95% C.I.:62.817-97.537), while it was calculated as79.204 months (95%C.I.:66.386~92.022)for EC-negative group. Please refer to **Table 5** and **Figure 2B** for details.

**Table 5:** Survival status and time analysis in EC-positive group and EC- negative group.

EC	Median overall survival	S.E. (95% CI)	Log-Rank	P
Negative group	79.204	6.54(66.386-92.022)	0.008	0.928
Positive group	80.177	8.857(62.817-97.537)		
Total	79.703	5.339(69.238-90.169)		

S.E., standard error; CI, confidence interval; P, p-value; EC, endometriosis.

After stratifying selected independent predictors, the effects of endometriosis on PFS and OS were also analyzed (see **Table 6**). The conclusion was that the two cohorts had no statistical significance in PFS after stratification for chemotherapeutic effect (Chemotherapy sensitivity: p-value=0.8354; Chemotherapy resistance: p-value=0.5275) or FIGO stage in OS after stratification (Stage I: p-value=0.8172; Stage II-IV: p-value=0.1276) or chemotherapeutic effect (Chemotherapy sensitivity: p-value =0.6425; Chemotherapy resistance: p-value=0.1349).

**Table 6:** Survival analysis after stratification for independent predictors.

Parameter	EC (%)		Log-Rank	P
	(-)	( +)		
OS				
Chemotherapy sensitivity	120(81-120)	/	<0.0001	0.6425
Chemotherapy resistance	24(18-43)	20	<0.0001	0.1349
FIGO stage				
Stage I	120(60-120)	/	<0.0001	0.8172
Stage II-IV	48(38-87)	36(20-43)	<0.0001	0.1276
PFS				
Chemotherapy sensitivity	110(46-117)	/	<0.0001	0.8354
Chemotherapy resistance	3(1-4)	3(1-6)	<0.0001	0.5275

P, p-value; EC, endometriosis.

## Discussion

The relationship between endometriosis and OCCC has been extensively studied since Sampson first described EAOC in 1925, with multiple studies evaluating the incidence of endometriosis-related OCCC (17). Evidence suggests that women with endometriosis have a total ovarian cancer risk that is 1.2-1.8 times higher than that of the general population (18). Patients with such history are three times more likely to develop OCCC compared to those without it (19). A large-scale epidemiological study on over ninety-nine thousand women with endometriosis found an increased incidence rate of ovarian cancer among them (20). Herein, we examined 105 OCCC patients and investigated the effect of endometriosis as an independent prognostic factor. In this cohort, all patients were Han Chinese women, and approximately 42% of OCCC patients were endometriosis positive. After univariate, multifactorial, and stratified analysis of several confounding factors, we found that endometriosis had no significant impact on prognosis.

OCCC is a special type of EOC pathology characterized by unique clinical and molecular features (22, 27). However, not all women with OCCC have concomitant endometriosis at the time of diagnosis, further demonstrating that endometriosis-free OCCC and endometriosis-related OCCC seem to develop through different pathways. Although the association between ovarian endometriosis and OCCC has been established, the exact underlying molecular transformation mechanisms remain unclear (21). Some scholars believe that bcl-2 and p53 proteins may be related to the malignant transformation of endometrial cysts (42), and it is recommended to establish standards for identifying and monitoring risk factors in women with endometriosis, and to seek risk-reducing drug and surgical treatment options in these women (43).

Currently it is widely believed that patients with Endo-related OCCCs tend to be younger when diagnosed (28, 29); however our findings contradict those reports since our data shows no significant difference in age between groups (P<0.05) but rather an average 4-year younger age at onset for EC-positive group compared with EC-negative group. The reasons for this deviation may be due to limited pathological sampling after surgery where some patients who had concurrent internal heterotopic lesions were not clearly documented; or pathologists may overlook internal heterotopic lesions once they diagnose ovarian cancer or miss them due technical differences; Rapidly growing cancer cells may also destroy the original endometriotic lesions, thereby eliminating histological evidence of endometriosis. Due to conflicting data, further large-scale studies are needed to support the aforementioned viewpoint.

In addition, the single factor analysis in this study found that positive ascites cytology affected patients’ recurrence and survival time, which caused us to think about the current surgery for ovarian cancer. Currently there is no high-level evidence in evidence-based medicine to suggest that minimally invasive surgery has adverse effects (30, 31). Therefore, some clinicians choose laparoscopic surgery for ovarian cancer. In this study, a univariate analysis was used to statistically analyze the clinical factors related to patient recurrence and survival. The results showed that ascites and positive cytology of ascitic fluid were associated with patient recurrence and survival time, making them predictive factors affecting patient survival (P<0.001). However, in clinical practice, patients with endometriosis often present with significant symptoms such as dysmenorrhea and seek repeated medical treatment. Therefore, for early-stage clear cell carcinoma combined with endometriosis patients, a considerable number of physicians may initially diagnose it as benign conditions such as ovarian chocolate cysts and recommend laparoscopic exploration followed by removal of the affected ovary tumor for definitive diagnosis. Studies have shown that endometriosis leads to an average size of 15cm for OCCC ovarian masses which are predominantly multilocular and contain solid component (32, 33). For larger ovarian tumors accompanied by intrauterine adhesions due to endometriosis (34), there is a high risk of cyst rupture during laparoscopic surgery (35) leading to clinical stage upgrading and positive cytology of ascitic fluid which affects patient prognosis. Additionally, during laparoscopy procedures, instruments repeatedly entering contaminated incisions generate airflow and smoke from electrocoagulation or electrosurgery promoting dissemination and implantation of tumor cells; During laparoscopic surgery, filling the abdominal cavity with CO_2_ gas can cause diffuse damage to the entire abdominal cavity and facilitate the peritoneal dissemination of tumor cells (36–38). The head-down, feet-up position during laparoscopic surgery, once the tumor ruptures, can easily lead to tumor dissemination in the entire abdominal and pelvic cavities, including the upper abdomen.

Moreover, OCCC has a low response to chemotherapy and exhibits some degree of drug resistance (39), thereby increasing the likelihood of tumor recurrence. Therefore, in practical work, careful selection of suitable patients is necessary for laparoscopic surgery to avoid iatrogenic tumor dissemination. Larger-scale studies are needed to validate the aforementioned observations regarding tumor rupture caused by laparoscopic and open surgeries in patients with stage IC1 OCCC.

Given the limited effectiveness of late-stage OCCC chemotherapy, the question of whether early-stage OCCC patients truly benefit from adjuvant chemotherapy has always been controversial. Multiple retrospective studies have shown that patients with advanced ovarian clear cell carcinoma (OCCC) have limited benefits from adjuvant chemotherapy, and there is no statistically significant difference in disease-free survival (DFS) and overall survival (OS) between patients who received chemotherapy and those who did not (41). However, there are also studies supporting the benefit of chemotherapy in early-stage OCCC patients. An analysis of 2,072 stage OCCC patients from the US National Cancer Database (NCDB) showed that those who received adjuvant chemotherapy had a higher 5-year OS than those who did not receive adjuvant chemotherapy(89.2% vs 86.2%, P<0.001) (40). After controlling for factors such as disease stage, age, race, hospital type, and comorbidities, adjuvant chemotherapy was associated with better OS. In this study, all patients were from the same medical center, so the treatment strategy was similar and the majority of patients received chemotherapy with a combination of paclitaxel and platinum drugs. We found no difference in chemotherapy efficacy between the two groups. However, through multivariate survival analysis, we discovered that the chemotherapy effect was an independent predictor of patient recurrence and survival outcomes (P<0.05). The risk of recurrence and death is higher in chemotherapy resistant patients compared to chemotherapy sensitive patients (HR= 5.596, 95%C.I.: 2.942 ~10.643).

In this study, we compiled comprehensive data analysis and reliable follow-up records from patients treated at our medical center with a unified clinical treatment approach. The study reveals the clinical characteristics, treatment features, and prognosis of ovarian clear cell carcinoma (OCCC) in China. Most patients were diagnosed at an early stage and received postoperative chemotherapy regardless of staging. Our findings indicate that endometriosis is not an independent prognostic factor for OCCC women. However, these findings are limited by the number of included patients and the retrospective design of the study, which may introduce selection bias. Therefore, considering the unique biological characteristics of OCCC and its increasing incidence rate, especially in East Asia, larger-scale well-designed prospective studies are still needed to establish a stronger connection between endometriosis and OCCC.

## Data Availability

The raw data supporting the conclusions of this article will be made available by the authors, without undue reservation.
